# Reply to the commentary of Burkhard Madea and Elke Doberentz on our article “An assessment of the Henssge method for forensic death time estimation in the early post-mortem interval”

**DOI:** 10.1007/s00414-025-03437-x

**Published:** 2025-02-04

**Authors:** Fabian Heinrich, Benjamin Ondruschka, Sven Anders-Lohner

**Affiliations:** 1 Institute of Legal Medicine, University Medical Center Hamburg-Eppendorf, Hamburg, Germany; 2 Department of Medical Statistics, London School of Hygiene and Tropical Medicine, London, UK; 3 Centre for Data and Statistical Science for Health, London School of Hygiene and Tropical Medicine, London, UK

We thank the authors for their interest in our study [1] and their commentary.

A predictive model is developed to forecast an outcome of interest within a target population based on the characteristics of the cases. The model aims to identify key factors associated with the outcome of interest to achieve a high degree of agreement between observed and predicted outcome values (i.e. model precision). For models that seek to predict the time since death, the target population includes individuals who died at a crime scene, as predicting the time since death is especially relevant in these instances. The Henssge model, based on the work of Marshall and Hoare [2], was developed using a cohort of individuals who were not reported to have died at a crime scene and for whom a known time since death was established, comprising a convenience sample of 39 bodies from corpses admitted to an Institute of Legal Medicine in Germany (cases of sudden death in public an hospital cases [3, 4]). Furthermore, model development was conducted without external validation steps, making it prone to overfitting.

Here, we provide external validation of the Henssge model under comparable conditions. We enrolled a cohort of individuals who were reported not to have died at a crime scene and for whom a known time of death had been established. This cohort comprises a convenience sample of 76 bodies from individuals admitted to the Institute of Legal Medicine in Hamburg. Consequently, the transportability of the Henssge model to this cohort should be notably high. However, even when assessing the Henssge model within the subgroup of patients stored at room temperature, where the assumption of a constant ambient temperature likely applies, we observe moderate agreement between the observed and predicted time since death. Indeed, this moderate agreement reported in the observed and predicted time since death is not particularly surprising, as it has already been noted by colleagues from Jena [5].

According to the Henssge model, the time interval between death and admission was intentionally overlooked. The ambient temperatures during storage, along with the unclothed and uncovered conditions of individuals from admission to measurement, were extrapolated to this timeframe. This extrapolation serves as a valid approximation of reality only if the ambient storage temperature from admission to measurement reflects that from death to admission and the clothing and covering conditions of individuals from admission to measurement align with those from death to admission. The accuracy of this assumption remains uncertain for both studies [1, 3]. Considering this extrapolation, our study found the model performance to depend on the time elapsed between death and admission.

During our investigation, we intentionally most likely modified the ambient storage temperature in the group of cold-stored cases. We demonstrated that in scenarios where the assumption of a constant ambient temperature and the state of clothing and coverings is likely violated - given that most individuals reported to have died within a specific timeframe died at room temperature - the concordance between the predicted and observed time since death decreases. The poor performance of the model in cold-stored cases and across the overall cohort can be partly attributed to the violation of the assumption of a constant ambient temperature and the state of clothing and coverings from death until measurement. We reported stratified estimates for a meaningful interpretation of agreement probabilities. Non-random variations in the ambient storage temperature between approx. 9 and 17 °C after admission to the institute have also been described by Henssge for his sample. The former non-random variations in ambient temperature similarly violate the assumption that ambient storage temperature remains constant.

While we observe moderate model performance even within a cohort where the model’s transportability should be exceptionally high, its transportability to individuals who died at a crime scene may be even lower for several reasons. First, a different range of ambient temperatures can be encountered at a crime scene, including sub-zero temperatures rarely observed in the cohort from which the model was developed. Therefore, predictions from the model under such circumstances rely on extrapolations beyond the data. Secondly, several factors not accounted for during model development may significantly affect the observed cooling kinetics, further compromising the model’s transportability. Among others, polytrauma and significant blood loss may influence the cooling kinetics and might lead to even poorer model performance in individuals dying at a crime scene.

Literature assessing model performance on cohorts of individuals who died at crime scenes has consistently encountered notable limitations. Most authors compared the police-investigated interval for the time since death with the 95% prediction interval for the same (for details, see [1]). However, this often leads to only limited conclusions about whether the actual time since death aligns with the 95% prediction interval. Notably, it is feasible to construct scenarios where the 95% prediction interval for the time of death and the time since death intervals defined by police investigations overlap, yet the actual time of death may not correspond with the 95% prediction interval. Furthermore, the time since death interval defined by police investigations is not a statistical interval but rather a range of plausible values determined empirically. As a result, conducting standard hypothesis testing on these intervals is inappropriate and may yield unreliable conclusions.

In summary, the Henssge model was neither developed using a cohort of cases from crime scenes nor did we assess the model for its applicability to such a cohort. However, consistent with previous findings [5], we observed only moderate agreement between the actual and predicted time since death in a group of corpses with a known time since death, where the applicability of the Henssge model should be exceptionally high. The applicability of the Henssge model to individuals who die at a crime scene requires further evaluation due to various statistical deficiencies in the literature regarding model performance assessment within those cohorts. A future model should encompass more cases, examine factors associated with predicting the time since death, and integrate internal and external validation steps to prevent overfitting while striking a balance between high model complexity that reflects reality and a parsimonious, forensically feasible model.
